# Spatial and Temporal Trends in the Invasion Dynamics of the Ring-Necked Parakeet (*Psittacula krameri*) in the Urban Complex of Thessaloniki, Greece

**DOI:** 10.3390/ani16020224

**Published:** 2026-01-12

**Authors:** Charalambos T. Thoma, Konstantina N. Makridou, Dimitrios E. Bakaloudis

**Affiliations:** Laboratory of Wildlife and Freshwater Fisheries, School of Forestry and Natural Environment, Aristotle University of Thessaloniki, P.O. Box 241, 541 24 Thessaloniki, Greece; makridkn@for.auth.gr (K.N.M.); debakaloudis@for.auth.gr (D.E.B.)

**Keywords:** *Psittacidae*, dynamic occupancy, N-mixture models, invasive species, urban ecology, population growth

## Abstract

Invasive alien species pose a major threat to global biodiversity, especially within Europe. Understanding their spatial and temporal dynamics is essential for effective management planning and implementation. The ring-necked parakeet (*Psittacula krameri*, hereafter RNP) has been established in Greece for over four decades, yet its invasion dynamics remain unstudied despite pilling evidence of ecological impacts. During 2024 and 2025, we conducted repeated transect surveys across 99 1 km^2^ grid squares within the urban complex of Thessaloniki to assess environmental factors influencing occupancy and abundance, and to estimate RNP population trends. Dynamic occupancy and N-mixture models revealed that both the presence and abundance of RNP were positively associated with the proportion of dense urban fabric and urban green areas. The proportion of occupied sites increased by more than 10% between survey years (2024–2025), while the estimated population growth rate for this interval was 1.64, signaling a substantial short-term increase. Our findings provide the first detailed evidence of an established and growing RNP population within the urban complex of Thessaloniki, Greece. Continued monitoring and research on ecological impacts are essential, while any management actions should be developed with public engagement to ensure social acceptance and long-term effectiveness.

## Simple Summary

The ring-necked parakeet is a charismatic parrot that has become one of the most successful invasive bird species in Europe. Originally from Africa and Asia, its presence in Greece counts more than four decades. However, until now, no one has studied how its population is changing or what makes it thrive in Greek cities. In this study, we recorded the presence and abundance of parakeets across the urban complex of Thessaloniki to understand where they live, how many there are, and how their presence and numbers are changing. We found that their presence is mostly associated with dense urban areas and green spaces. In addition, their population showed strong short-term growth, increasing by an estimated 64% from 2024 to 2025. Our results suggest that Thessaloniki supports a large and expanding parakeet population. Keeping track of their numbers in the future is important, since their spread could affect native wildlife.

## 1. Introduction

Human activities during the last two centuries have led to a significant increase in the intentional or unintentional translocation of species beyond their native ranges [1,2,3]. While many introduced species fail to persist, some establish viable populations, whilst an even smaller portion is able to spread and cause diverse negative impacts [4]. Such species are labeled invasive [4] and are considered as one of the leading causes of biodiversity loss worldwide [5,6]. In Europe, the rate of invasion by non-native species is expected to rise in the near future [5]. Therefore, systematic monitoring of established non-native populations [7], along with an understanding of the mechanisms driving invasion dynamics in space and time [8] are essential for safeguarding biodiversity and reducing the impacts of native taxa [9].

Birds are among the most frequently introduced animal groups [2], with at least 971 species recorded outside their native ranges [2] and approximately 420 now supporting self-sustaining populations [2]. Among bird species, parrots and parakeets illustrate a high invasion and establishment success [9,10], mainly due to their popularity in the pet trade [11] and their phenotypic plasticity [12]. As a consequence, more than 16% of the world’s 352 parrot species have established breeding populations in non-native environments [11]. Similarly, in Europe 53 non-native parrot species have been recorded, of which 12 have established viable populations [13].

Originally distributed across Africa and Asia [14], the ring-necked parakeet (*Psittacula krameri*, hereafter RNP) is now one of the most successful and widespread introduced parrot species in Europe and the Mediterranean basin [7]. By 2015, its European population numbered at least 85120 individuals [7], while the species has been recorded in 92 countries beyond its native range [15]. Within Europe, the species occurs predominantly in major urban centers, where it shows a strong preference for densely inhabited areas and urban green spaces [10,16,17,18,19].

Listed as one of the 100 most harmful alien species in Europe [20], the RNP affects native ecosystems and human activities throughout its European invaded range [21]. The species is primarily recognized as a nesting competitor of native fauna [22,23,24,25,26]. Other impacts on native species include foraging interference [27], as well as mobbing [28] and fatal attacks [22,29,30]. Additional negative impacts reported in the literature include agricultural damages [31], acoustic disturbance [32], and possible disease transmission [31]. Despite rising evidence on the negative impacts related to the RNP, their magnitude; especially on native fauna, remains controversial [33,34]. Moreover, efforts to control the species are seldomly initiated due to a general lack of fundamental information [18], as well as due to the general positive public perception of this charismatic species [35].

In Greece, published information regarding the species is very limited. According to Pârâu et al. [7], Greece is home to 1000 individuals (800–1200), most of them found in the city of Athens. Other, more recent media sources report a population of 2000–3000 individuals, however no details regarding these estimates are provided. It is believed that the species was initially introduced in the 1980s through the pet trade and became established by 1990, following an accidental release. Despite its presence for over 40 years, the species’ ecology and invasion dynamics have never been studied before.

Given the species’ exponential increase in population numbers and rapid expansion potential [7], as well as the rising evidence of negative impacts on native fauna [31], managers; primarily the Ministry of Environment and Energy in Greece, together with regional authorities, Forestry Services, and protected-area management units, are often called upon to take effective measures aimed at reducing invasive species’ population numbers or controlling their spread [36]. Monitoring and comprehending the spatial and temporal trends of invasive species is key to understanding and managing biological invasions successfully [37,38,39].

In this context, our study offers the first detailed analysis of the spatial and temporal trends of RNP expansion in Greece, focusing on the urban complex of Thessaloniki. Based on previous studies regarding the species, we hypothesized that (i) RNP occupancy and abundance would be positively associated with urbanization intensity and greater availability of nesting and foraging sites, and (ii) the population would show an increasing trend between survey years, reflecting the species’ ongoing spread and expansion. Our study could provide insights into the mechanisms that facilitate RNP establishment and persistence in Mediterranean urban environments and could serve as a baseline for long-term monitoring of the species’ spread and population size, and for assessing the outcomes of management and control measures.

## 2. Materials and Methods

### 2.1. Study Area

**Figure 1:**
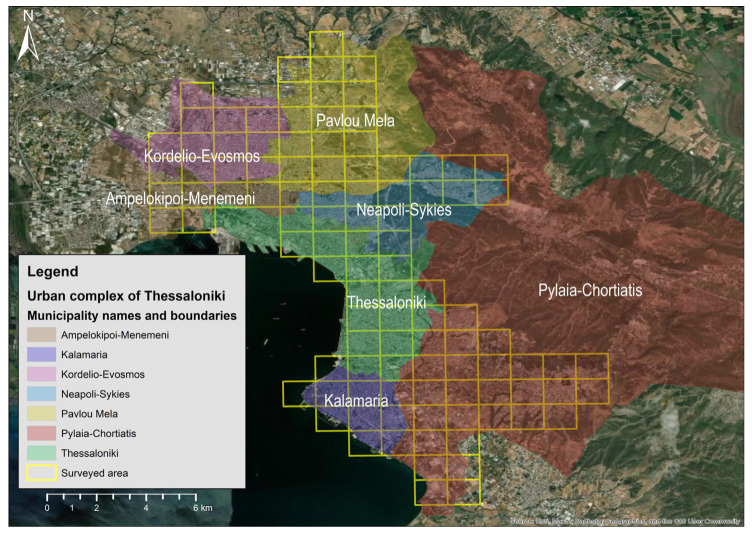
Depiction of the seven municipalities forming the urban complex of Thessaloniki, Greece. Yellow grid squares (*n* = 103) represent the selected sites for potential surveying of the ring-necked parakeet occupancy and abundance.

Thessaloniki is located in northern Greece, within the region of Central Macedonia, and is the country’s second-largest urban center after Athens. The city forms part of the Metropolitan Unit of Thessaloniki (MUTH), an administrative subdivision of the region that includes 13 municipalities [40]. The main urban complex consists of seven contiguous municipalities; Thessaloniki, Ampelokipoi-Menemeni, Kalamaria, Kordelio-Evosmos, Neapoli-Sykies, Pavlos Melas and Pylaia-Chortiatis, which together make up the city’s continuous built-up area (Figure 1). Geographically, the city is bordered to the north by the Seich Sou peri-urban forest, to the south by Thermaikos gulf, to the west by the industrial zone of Sindos and the port area, and to the east by suburban municipalities and open landscapes extending toward Thermi and the Halkidiki peninsula. The urban complex of Thessaloniki concentrates most of the population of the wider metropolitan area. The MUTH has a total population of about one million inhabitants [41] and covers approximately 1285 km^2^, serving as the main economic and cultural center of northern Greece. The city experiences a temperate Mediterranean climate, characterized by hot, dry summers and mild, wet winters, although notable seasonal variations may occur [42]. The broader area is characterized by high floristic [43] and faunal diversity [44,45].

### 2.2. RNP Surveys

Data describing the presence and abundance of RNP were obtained from field surveys conducted during what is believed to be the species’ breeding period. The study area was divided into 1 km^2^ grid squares [46], from which 103 were selected for potential surveying (Figure 1). The survey was designed to cover all of the municipalities that form the urban complex of Thessaloniki. Grids were selected based on accessibility and potential suitable habitat for RNP; however, four of the selected grid squares were ultimately not surveyed due to access restrictions or safety concerns.

Within each grid square, we carried out line transect surveys [47,48]. Transects mainly followed residential roads, pedestrian tracks or dirt roads and had a mean length of 1184 m (range: 492–2075 m). Each transect was walked at a slow pace by a single observer and all RNP seen or heard along each transect were recorded. RNP auditory cues primarily guided visual searches. When visual confirmation was not possible, vocalizations originating from a single point source were attributed to one individual. Individuals flying over the area during surveys were excluded.

Surveys were conducted during two consecutive primary sampling periods, corresponding to May 2024 and May 2025. Within each primary period, each transect was surveyed twice, resulting in two repeat surveys per site per year. Repeated surveys were carried out within the same month to satisfy the assumption of population closure within primary periods. Surveys were scheduled in two daily time windows (06:30–09:30 and 17:00–19:30) and each transect was surveyed once in each time window per year, ensuring temporal variation in detectability was accounted for [46]. Surveys were carried out during days with good weather conditions (no rain, low wind).

This level of within-season replication represents a deliberate trade-off between temporal and spatial sampling effort. Given the conspicuous and vocal behavior of RNP in urban environments and the moderate to high detection probability observed in our study, two-repeat surveys per site are considered sufficient and can provide reliable estimates [49,50], while allowing broader spatial coverage [51].

### 2.3. Environmental Variables

To better understand the associations between landscape features and RNP occupancy and abundance, we derived a set of variables describing land cover characteristics, anthropogenic influence and topography (Table S1). Candidate variables were selected based on previously documented effects of these factors on invasive parakeet distribution and abundance [10,19,52,53]. All variables were cropped to match the extent of the survey area and were resampled to a 1 km resolution using ArcGIS 10.2. Prior to any analyses, we assessed the linear correlation among all candidate variables and discarded those with a correlation coefficient greater than 0.6 [54].From an initial set of 14 candidate variables [55,56,57,58,59,60], six were retained for the final analyses and are described in Table 1.

### 2.4. Data Analysis

#### 2.4.1. Dynamic Occupancy Model

RNP detection and non-detection data were analyzed using multi-season dynamic occupancy models [61], wherein initial occupancy (ψ_1_; i.e., the probability that a site was occupied during the first year of surveys), colonization (γ; i.e., the probability that an unoccupied site becomes occupied between survey years), extinction (ε; i.e., the probability that an occupied site becomes unoccupied between survey years), and detectability (*p*; i.e., the probability of detecting the species during a survey, conditional to its presence) are jointly estimated to describe state transitions across the surveyed area. Time of survey, date and year, as well as line transect length (Table 1), were considered as potential covariates affecting detectability. All environmental variables (Table 1) were used to model the probability of initial occupancy (*ψ*_1_), as well as colonization probability (*γ*). Finally, because no instances of local extinction were detected during the second survey year (i.e., all sites occupied in 2024 remained occupied in 2025), extinction probability (ε) was held constant throughout the modeling process. This constraint reflects the observed biological pattern during the study period and ensures model estimability. All variables were z-standardized prior to model fitting, so that their relative effect could be compared in model outputs. Model development followed a sequential approach [8], beginning with the estimation of *p*, while keeping all other parameters constant (Table S2). We then used the top ranked *p* model to develop a set of models examining *ψ*_1_, while keeping *γ* and *ε* constant (Table S3). Sequentially, we used the top ranked *ψ*_1_ model from the previous step to create a list of models examining *γ*, while keeping *ε* constant (Table S4). The top ranked model from this step was selected as the final model on which the interpretation of our results was based. Model fit of the final model was tested using a parametric bootstrap goodness-of-fit test based on Pearson’s *χ*^2^, where *p* > 0.05 indicates adequate model fit [62,63]. Dynamic occupancy was investigated via the *colext* function of the *unmarked* package [63] in R [64].

#### 2.4.2. Dynamic N-Mixture Model

RNP population changes between 2024 and 2025 were estimated using the models of Dail and Madsen [65]; also known as dynamic N-mixture models, which generalize the [66] N-mixture model by relaxing the population closure assumption. In each dynamic N-mixture model, four distinct parameters are estimated describing population and observation processes: initial abundance (*λ*_1_), recruitment (*γ*) and apparent survival (*ω*), and detection probability (*p*). Count data were analyzed using the *unmarked* package [63] in R [64], and models were fitted with the *pcountOpen* function. For all models, we set the K-value to 50, which was more than double the highest site count (*n* = 21 individuals) and trials showed it was sufficient not to influence parameter estimates. Model fitting followed a two-stage approach. In the first stage, we built a set of candidate models to estimate p, using the same variables that could potentially affect detectability as in our dynamic occupancy models (Table 1) and explored three alternative statistical distributions; Poisson (P), Negative Binomial (NB), and Zero-Inflated Poisson (ZIP). The top ranked *p* model (Table S5) then served as the basis for the subsequent models for *λ*_1,_ in which the environmental variables (Table 1) were used as potential covariates affecting abundance. The top ranked model (Table S6) from this step was selected as the final model on which the interpretation of our results was based. In all models, recruitment rate (*γ*) and apparent survival (*ω*) were kept constant and model dynamics were set to “*trends*”. Model fit of the final model was tested using a parametric bootstrap goodness-of-fit test based on Pearson’s *χ*^2^, where *p* > 0.05 indicates adequate model fit [62,63].

For both the dynamic occupancy and the dynamic N-mixture models, parameter estimates with 95% confidence intervals not spanning zero were interpreted as highly informative, those with 85% confidence intervals not spanning zero were interpreted as moderately informative, and those with 85% confidence intervals spanning zero were considered uninformative [67].

## 3. Results

**Figure 2:**
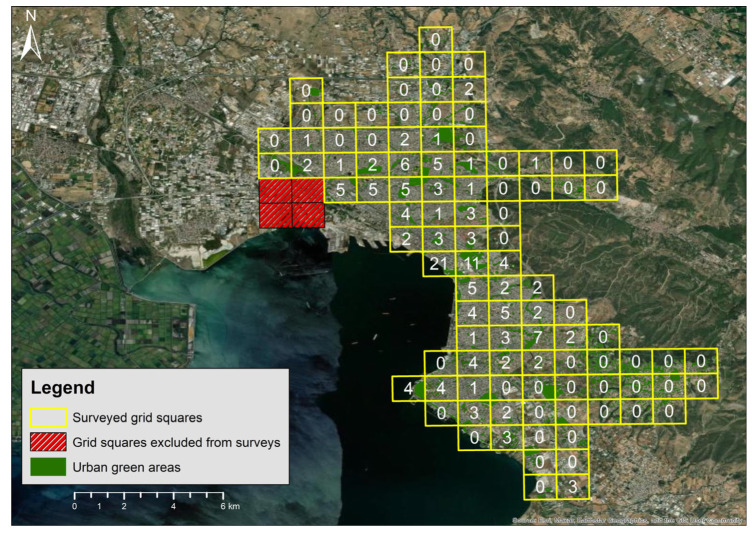
Maximum number of ring-necked parakeets recorded across four visits in each grid square surveyed in May 2024 and May 2025. Red squares depict un-surveyed grids, whereas urban green areas are shown in green.

Of the 99 grid squares that were surveyed during both breeding periods, RNPs were detected at least once in 45 of them (Figure 2). Naïve occupancy (i.e., without accounting for imperfect detection) illustrated a 11.2% increase between survey years, from 34.3% in 2024 to 45.5% in 2025. At sites where the species was detected at least once, the mean observed detection frequency across surveys was 0.72 (range: 0.25–1). Similarly, observed abundance of RNP varied among sites and survey years. Across all surveyed grid squares, the maximum number of individuals recorded at a single site (i.e., based on the higher count from the two surveys conducted within each year) increased from 97 individuals in 2024 to 156 individuals in 2025 (Figure 2). A small number of sites, located within the city center, supported particularly large aggregations. At sites were RNP were detected at least once, the mean naïve abundance per site increased from 2.85 individuals (range: 1–15) in 2024 to 3.46 individuals (range: 1–21) in 2025.

**Figure 3:**
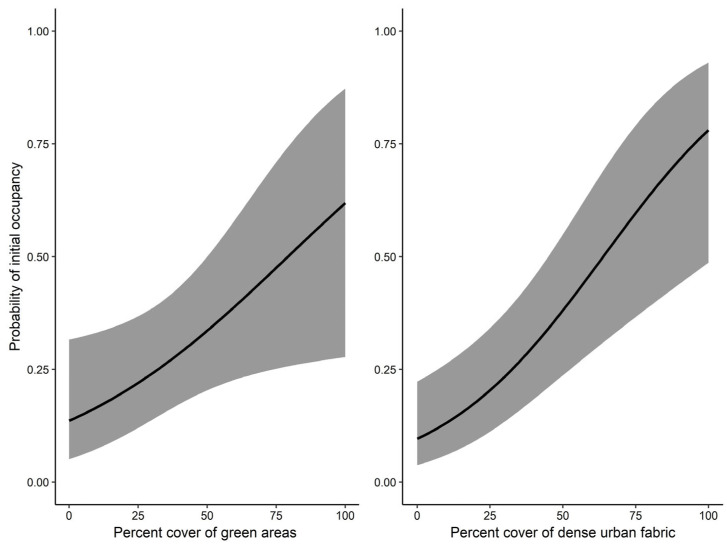
Predicted relationships between percentage cover of green areas and dense urban fabric and the probability of initial occupancy by ring-necked parakeet in the urban complex of Thessaloniki, Greece. Shaded areas represent 95% confidence intervals of the estimations.

Our top dynamic occupancy model (Table 2) showed that detection probability (*p*) was not influenced by any of the observation-level covariates. The null detection model provided the best fit, illustrating a detection probability of 78.7% ($\hat{\beta }$ = 1.31, S.E. = 0.217). The estimated probability of initial occupancy (*ψ*) across the study area was 25.5% ($\hat{\beta }$ = −1.07, S.E. = 0.338). Initial occupancy was positively associated with the percentage cover of green areas (PC_Green: $\hat{\beta }$ = 0.778, S.E. = 0.366) and dense urban fabric (PC_Urban: $\hat{\beta }$ = 1.17, S.E. = 0.322) (Figure 3). Both these variables were highly informative, as their 95% confidence intervals did not span zero. Conversely, elevation (DEM) illustrated a negative association ($\hat{\beta }$ = −1.024, S.E. = 0.473), however this variable was uninformative. The probability of a grid square being colonized by the species increased with increasing cover of dense urban fabric (PC_Urban: $\hat{\beta }$ = 4.63, S.E. = 3.04). This variable was moderately informative, as its 85% confidence intervals did not overlap zero. Over the same study period, the estimated probability of colonization (γ) was 8.63% ($\hat{\beta }$ = −2.36, S.E. = 1.14), while extinction probability (ε) was effectively zero ($\hat{\beta }$ = −10.5, S.E. = 33.6). Colonization probability tended to decrease with increasing distance from arable land (Dis_Arable: $\hat{\beta }$ = −2.24, S.E. = 1.84), although this effect was uninformative. The final dynamic occupancy model from which the results were drawn had adequate model fit according to Pearson’s χ^2^ (*p* = 0.72).

**Figure 4:**
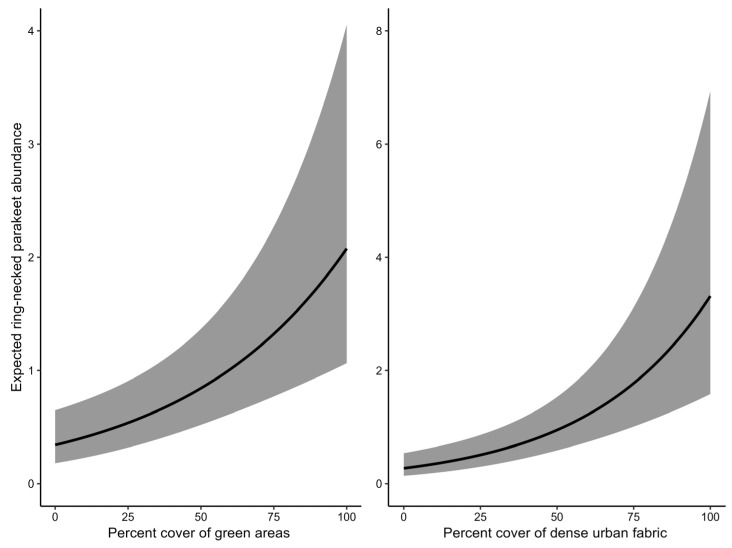
Predicted relationships between percentage cover of green areas and dense urban fabric and the probability of initial abundance of the ring-necked parakeet in the urban complex of Thessaloniki, Greece. Shaded areas represent 95% confidence intervals of the estimations.

For the best supporting dynamic N-mixture model (Table 3), the negative binomial distribution provided a better fit. Detection probability (*p*) was not influenced by any of the observation-level covariates, with the null detection model providing the best fit. Detectability was estimated at 43.8% ($\hat{\beta }$ = −0.254, S.E. = 0.213). The model estimated a mean initial abundance (λ_1_) of 0.62 individuals per grid square in the first survey year ($\hat{\beta }$ = −0.472, S.E. = 0.255), representing baseline abundance at average covariate values. Initial RNP abundance (λ_1_) was positively influenced by the percentage cover of green areas (PC_Green: $\hat{\beta }$ = 0.602, S.E. = 0.150) and dense urban fabric (PC_Urban: $\hat{\beta }$ = 0.835, S.E. = 0.179) (Figure 4), but showed a negative association with elevation (DEM: $\hat{\beta }$ = −1.270, S.E. = 0.303). The 95% confidence intervals for the first two variables did not span zero, indicating high certainty regarding their effect on initial abundance. Predicted RNP abundance during the first breeding period was 183 (range: 110–356) individuals, and increased to 304 (range: 199–468) individuals during the second year of surveys. The estimated growth rate (λ) between survey years was 1.64 (1.37–1.97), reflecting a strong population increase of 64% from 2024 to 2025. The best supporting dynamic N-mixture model from which the results were drawn, illustrated adequate model fit according to Pearson’s χ^2^ (*p* = 0.174).

## 4. Discussion

Our study provides clear evidence that the urban complex of Thessaloniki supports a thriving and expanding population of RNP. The key findings of widespread occupancy and strong positive growth rate during our study period, highlight the remarkable ability of this non-native species to flourish in urban-dominated environments. We found a consistent and positive association between RNP populations and two key urban land cover types; dense urban fabric and urban green areas. This pattern was consistent across both the dynamic occupancy and N-mixture best supporting models, indicating that these landscape features not only increase the likelihood of the species being present in an area, but also support higher local abundances.

Urban areas have become the primary habitat for RNPs, as well as other non-native parakeet species, especially throughout Europe [7,9,10,17]. Large urban areas are generally warmer than surrounding rural areas [68], due to the heat-island phenomenon [69]. Although the RNP is an adaptable species capable of tolerating a range of environmental conditions [70], low temperatures can limit its ability to establish viable populations [19]. In fact, extreme cold weather has been documented to have caused local population declines or extinctions [71,72]. Moreover, urban settings generally provide plentiful feeding opportunities year-round, supported by the wide variety of both native and ornament vegetation found in urban green areas, streets and private gardens [73,74,75,76]. In Greece, RNPs have been observed feeding on a variety of plant species, including native trees such as *Cupressus sempervirens* and *Olea europaea*, as well as introduced species like *Melia azedavach* and *Phoenix canaviensis* [77]. This dietary breadth reflects the species’ opportunistic and adaptable feeding strategy which allows it to exploit a variety of food sources, including those that may be toxic to other animals [77].

Besides providing food resources, urban green areas may also contribute to the availability of nesting and roosting sites across the urban landscape [19,78]. Mature trees, commonly retained for their visual or cultural value [78], are particularly valuable to RNPs, as these provide abundant cavities for nesting and sheltered spots for roosting [18,19,26,79]. However, when natural tree cavities are limited, RNPs may exploit alternative nesting sites such as cavities in old buildings [16], or in some cases they may even excavate their own nesting cavities [25,80], highlighting once again their behavioral plasticity.

While nest site availability may act as a limiting factor [81], several studies have found an exponential increase in RNP numbers across its invaded range. For example, a study in Italy found a 70.5% annual increase in ring-necked population numbers [33]. Similarly, a mean annual increase of ~62% and ~63% were found in Spain and Turkey, respectively [17,82]. In the UK, RNP population numbers have increased by 2406% since 1995 [83], whereas in the Netherlands there was an 1582% increase in breeding pairs since 1998 [84]. Similar patterns have also been reported for France, Belgium and Germany [7]. In Greece, the total number of RNPs remains somewhat unknown. Pârâu et al. [7] report a lower estimate of 1000 individuals, while several media sources report an estimate of 2000 to 3000 individuals. Our study provides the first ever estimate for both the spread and abundance of the species within Greece. Our two-year data indicate a substantial population increase, similar in magnitude to growth rates observed during establishment phases in other regions. This recurring pattern may be attributed to the species’ high reproductive success [85].

Our results indicate a substantial RNP population within the study area, although the actual number of individuals is likely higher. It is likely that additional colonies exist outside our surveyed area. In fact, several individuals were observed up to ~10 km beyond the survey boundaries (near the thermal springs of Lagkadas, personal observation), indicating that the species may be more abundant and widespread than our data suggest. Furthermore, the relatively low detection probability (~40%) estimated by the N-mixture model may also imply that not all individuals were detected during counts. While the species is highly vocal [32], and its presence can be easily detected, the species’ predominantly green plumage likely provides effective camouflage in tree canopies, making individual birds difficult to distinguish. In fact, this could perhaps explain the high detection probability (~80%) in our dynamic occupancy model, in relation to the N-mixture model. Hence, conducting roost counts, particularly in winter when deciduous trees have reduced foliage, may improve detectability and produce more accurate abundance estimates, given that all major roost sites are previously identified.

Nevertheless, monitoring the spatial and temporal trends of invasive species constitutes a fundamental tool, based on which long term management initiatives can be developed [36,46]. However, implementing management actions for RNP may be particularly challenging due to two key factors: its high reproductive capacity [85], and public opposition [86,87]. In our study, population numbers increased exponentially, while naïve occupancy increased by more than 10% in between surveys, highlighting the species’ rapid expansion potential. Such population dynamics, along with the species’ high reproductive potential and behavioral flexibility, can hinder large-scale or long-term management efforts, as populations may quickly recover following control interventions. Conversely, control or eradication has been proven successful in some cases. For instance, Anderson et al. [88] successfully reduced the numbers of established RNPs on the island of Kaua’I and concluded that managers should consider long-term, alternative management options. Similarly, eradication has been successful only for small or developing insular populations [36,89]. In all cases, RNP culling was performed by either shooting or a combination of trapping and shooting. However, in densely populated urban areas such as Thessaloniki, these methods may be unsuitable mainly due to the potential public safety risks associated with shooting, and strong public opposition to such practices.

Public perception plays a crucial role in the management of invasive species [32,34,86,87]. The RNP is often viewed positively by the public due to its charismatic appearance [90], which can hinder the implementation of any control measures, unless accompanied by well-designed awareness and outreach campaigns [91]. As such, any future interventions should be planned in conjunction with transparent communication and consultation with the public, in an effort to minimize opposition [35,92]. Understanding the social drivers, values and perceptions underlying these disputes is essential for improving management acceptance and long-term effectiveness [34,86,87].

Although there is currently no mandatory requirement to control RNP populations in Europe [11], their strong population increase observed during the study and high modeled growth potential may pose risks to native species mainly through competition for resources [18,22,23,24,25,27,93]. To anticipate and mitigate these risks, regular monitoring of population status is essential [7], alongside targeted research on ecological impacts, and should be extended to suburban and rural areas beyond the urban core. Integrating these efforts with proactive management strategies and public engagement is crucial in ensuring informed, effective, and socially acceptable conservation decisions.

## 5. Conclusions

Our study demonstrates that RNPs occupancy and abundance are positively associated with dense urban areas combined with green spaces. This pattern is also reflected in both its widespread distribution and the substantial population growth we documented within the urban complex of Thessaloniki between 2024 and 2025. Our findings indicate a strong potential for continued expansion across the urban landscape. Hence, regular monitoring of population trends and distribution is essential for identifying emerging impacts on native species and for supporting timely management decisions. Any future control measures should be developed in close collaboration with the public to ensure social acceptance and long-term effectiveness.

## Figures and Tables

**Table 1:** List of environmental and observation level (detection probability) variables used to model ring-necked parakeet dynamic occupancy and abundance.

Parameter: Initial Occupancy (ψ_1_), Colonization (γ), Initial Abundance (λ_1_)			
Acronym	Description	Units	Source
PC_Urban	Percentage cover of dense urban fabric (estimated from Urban Atlas Land Cover class 11,100: Continuous urban fabric (S.L.: >80%))	%	[55]
PC_Forest	Percentage cover of forests (estimated from Urban Atlas Land Cover class 31,000: Forests)	%	[55]
PC_Green	Percentage cover of urban green areas (estimated from Urban Atlas Land Cover classes 14,100: Green urban areas, 12,100: Military and public units (only abandoned military units and university campus were included) merged with Urban Atlas Street Tree Layer	%	[55,56]
Dis_Arable	Distance (measured from the center of each grid square) to the nearest arable land	m	[55]
GHII *	Global Human Influence Index	-	[57]
DEM	Elevation	m	[58]
**Parameter:** Detection probability (*p*)			
**Acronym**	**Description**	**Units**	**Source**
mas	Time of survey expressed as minutes after sunrise	Integer	-
Jday	Date of survey expressed as Julian date	Integer	-
Year	Year of survey	Integer	-
LT	Line transect length	m	-

* GHII is a global index that measures cumulative human pressure based on population density, land use, infrastructure, and accessibility.

**Table 2:** Results of the final dynamic occupancy model for the ring-necked parakeet in the urban complex of Thessaloniki, Greece.

Probability of	Variable	$\mathrm{Parameter}\;\mathrm{Estimates}\;(\hat{\beta })$	SE
Initial occupancy (logit-scale)	(*Intercept*)	−1.07	0.338
	*DEM*	−1.02	0.473
	*PC_Green* *	0.78	0.366
	*PC_Urban* *	1.17	0.322
Colonization (logit-scale)	(*Intercept*)	−2.36	1.14
	Dis_Arable	−2.24	1.84
	PC_Urban **	4.63	3.04
Extinction (logit-scale)	(*Intercept*)	−10.5	33.6
Detection (logit-scale)	(*Intercept*)	1.31	0.217

* Indicates variables with 95% confidence intervals not spanning zero. ** Indicates variables with 85% confidence intervals not spanning zero.

**Table 3:** Results of the final dynamic N-mixture model for the ring-necked parakeet in the urban complex of Thessaloniki, Greece.

Parameter	Variable	$\mathrm{Parameter}\;\mathrm{Estimates}\;(\hat{\beta })$	SE
Initial abundance (log-scale)	(*Intercept*)	−0.472	0.255
	*DEM*	−1.270	0.303
	*PC_Green* *	0.602	0.150
	*PC_Urban* *	0.835	0.179
Growth rate (log-scale)	(*Intercept*)	0.498	0.092
Detection (logit-scale)	(*Intercept*)	−0.254	0.213
Dispersion (log-scale)	(*Intercept*)	0.227	0.33

* Indicates variables with 95% confidence intervals not spanning zero.

## Data Availability

Data are available from the corresponding author upon reasonable request.

## Supplementary Materials

The following supporting information can be downloaded at https://www.mdpi.com/article/10.3390/ani16020224/s1: Table S1: Description of all environmental variables that were initially considered in modeling the dynamic occupancy and abundance of ring-necked parakeet (*Psittacula krameri*) within the urban complex of Thessaloniki, Greece; Table S2: Model results for probability of detection (*p*) from multi-season occupancy models of ring-necked parakeet in the urban complex of Thessaloniki, Greece, 2024–2025. Initial occupancy (ψ), colonization (γ), and extinction (ε) were held constant in all models. The models are ranked based on the lowest Akaike’s Information Criterion (AIC) where deltaAIC = AICi—minimum AIC, nPars = number of parameters, and AICwt = AIC weight. Only models with deltaAIC < 2 are shown; Table S3: Model results for probability of initial occupancy (ψ) from multi-season occupancy models of ring-necked parakeet in the urban complex of Thessaloniki, Greece, 2024–2025. The models included the top detection model (null), represented as *p* (top). Colonization (γ), and extinction (ε) were held constant in all models. The models are ranked based on the lowest Akaike’s Information Criterion (AIC) where deltaAIC = AICi—minimum AIC, nPars = number of parameters, and AICwt = AIC weight. Only models with deltaAIC < 2 are shown; Table S4: Model results for probability of colonization (γ) from multi-season occupancy models of ring-necked parakeet in the urban complex of Thessaloniki, Greece, 2024–2025. The models included the top detection model (null), represented as *p* (top) and the top initial occupancy model (DEM+PC_Green+PC_Urban), represented as ψ (top). Extinction (ε) was held constant in all models. The models are ranked based on the lowest Akaike’s Information Criterion (AIC) where deltaAIC = AICi—minimum AIC, nPars = number of parameters, and AICwt = AIC weight. Only models with deltaAIC < 2 are shown; Table S5: Model results for probability of detection (*p*) from dynamic N-mixture models of ring-necked parakeet in the urban complex of Thessaloniki, Greece, 2024–2025. Initial abundance (λ_1_), recruitment (γ), and apparent survival (ω) were held constant in all models. The models are ranked based on the lowest Akaike’s Information Criterion (*AIC*) where *deltaAIC* = AIC*_i_*—minimum AIC, *nPars* = number of parameters, and *AICwt* = AIC weight. Only models with deltaAIC < 2 are shown; Table S6: Model results for probability of initial abundance (*λ*_1_) from dynamic N-mixture models of ring-necked parakeet in the urban complex of Thessaloniki, Greece, 2024–2025. The models included the top detection model (null), represented as *p* (top) Recruitment (γ), and apparent survival (ω) were held constant in all models. The models are ranked based on the lowest Akaike’s Information Criterion (*AIC*) where *deltaAIC* = AIC*_i_*—minimum AIC, *nPars* = number of parameters, and *AICwt* = AIC weight. Only models with deltaAIC < 2 are shown.

## Abbreviations

The following abbreviations are used in this manuscript:

RNP	Ring-necked parakeet
MUTH	Metropolitan Unit of Thessaloniki
