# Supplementary material for: Spatial and Temporal Trends in the Invasion Dynamics of the Ring-Necked Parakeet (Psittacula krameri) in the Urban Complex of Thessaloniki, Greece
Source: Animals (Basel). 2026 Jan 12;16(2):224. doi: 10.3390/ani16020224 (PMC12837961; doi:10.3390/ani16020224)
Supplement: Supplementary file 1 [file animals-16-00224-s001.zip › animals-4062777-supplementary.pdf]

Table S1. Description of all environmental variables that were initially considered in modeling the dynamic occupancy and abundance of Ring-necked parakeet (*Psittacula krameri*) within the urban complex of Thessaloniki, Greece.

Category	Acronym	Description	Units	Type/resolution	Source
Land cover	PC_Urban	Percent cover of dense urban fabric (estimated from Urban Atlas Land Cover class 11100: Continuous urban fabric (S.L.: > 80%))	%	Vector	[55]
	PC_Open	Percent cover of open space (estimated from Impervious Built-Up class 0: Non-built-up)	%	Raster/10 m	[59]
	PC_Forest	Percent cover of forests (estimated from Urban Atlas Land Cover class 31000: Forests)	%	Vector	[55]
	PC_Herb	Percent cover of herbaceous vegetation (estimated from Urban Atlas Land Cover class 32000: Herbaceous vegetation associations)	%	Vector	[55]
	PC_Green	Percent cover of urban green areas (estimated from Urban Atlas Land Cover classes 14100: Green urban areas, 12100: Military and public units (only abandoned military units and university campus were included) merged with Urban Atlas Street Tree Layer	%	Vector	[55,56]
	PC_STL	Percent cover of Street Tree Layer	%	Vector	[56]
	NumP_STL	Number of discrete tree patches per grid square	Integer	Vector	[56]
	STL_Eden	Edge density of Street Tree Layer	m/km2	Vector	[56]
	Dis_ForHer	Distance (measured from the center of each grid square) to the nearest forest or herbaceous habitat	m	Vector	[55]
	Dis_Arable	Distance (measured from the center of each grid square) to the nearest arable land	m	Vector	[55]
	Dis_Green	Distance (measured from the center of each grid square) to the nearest urban green area	m	Vector	[55,56]
Topography	Dem	Elevation	m	Raster/ 25 m	[58]
Anthropogenic influence	GHII	Global Human Influence Index	-	Raster/ 1 km	[57]
	Road_den	Road density	m/km2	Vector	[60]

Table S2. Model results for probability of detection (*p*) from multi-season occupancy models of Ring-necked parakeet in the urban complex of Thessaloniki, Greece, 2024-2025. Initial occupancy (ψ), colonization (γ), and extinction (ε) were held constant in all models. The models are ranked based on the lowest Akaike's Information Criterion (*AIC*) where *deltaAIC* = AIC_i – minimum AIC, *nPars* = number of parameters, and *AICwt* = AIC weight. Only models with deltaAIC < 2 are shown.

Model	AIC	deltaAIC	nPars	AICwt
**ψ(.) γ(.) ε(.) *p*(.)**	**338,96**	**0,00**	**4**	**0,65**
ψ(.) γ(.) ε(.) *p*(jday)	340,18	1,22	5	0,35

*(.) indicates parameters that were held constant – no covariates effects

Table S3. Model results for probability of initial occupancy (ψ) from multi-season occupancy models of Ring-necked parakeet in the urban complex of Thessaloniki, Greece, 2024-2025. The models included the top detection model (null), represented as *p*(top). Colonization (γ), and extinction (ε) were held constant in all models. The models are ranked based on the lowest Akaike's Information Criterion (*AIC*) where *deltaAIC* = AIC_i – minimum AIC, *nPars* = number of parameters, and *AICwt* = AIC weight. Only models with deltaAIC < 2 are shown.

Model	AIC	deltaAIC	nPars	AICwt
**ψ(DEM+PC_Green+PC_Urban) γ(.) ε(.) *p*(top)**	**297,98**	**0,00**	**7**	**0,30**
ψ(DEM+PC_Forest+PC_Green+PC_Urban) γ(.) ε(.) *p*(top)	298,48	0,50	8	0,23
ψ(DEM+Dis_Arable+PC_Green+PC_Urban) γ(.) ε(.) *p*(top)	298,62	0,64	8	0,22
ψ(DEM+Dis_Arable+PC_Forest+PC_Green+PC_Urban) γ(.) ε(.) *p*(top)	299,52	1,54	9	0,14
ψ(DEM+GHII+PC_Green+PC_Urban) γ(.) ε(.) *p*(top)	299,83	1,85	8	0,12

*(.) indicates parameters that were held constant – no covariates effects

Table S4. Model results for probability of colonization (γ) from multi-season occupancy models of Ring-necked parakeet in the urban complex of Thessaloniki, Greece, 2024-2025. The models included the top detection model (null), represented as *p*(top) and the top initial occupancy model (DEM+PC_Green+PC_Urban), represented as *ψ*(top). Extinction (ε) was held constant in all models. The models are ranked based on the lowest Akaike's Information Criterion (*AIC*) where *deltaAIC* = AIC_i – minimum AIC, *nPars* = number of parameters, and *AICwt* = AIC weight. Only models with deltaAIC < 2 are shown.

Model	AIC	deltaAIC	nPars	AICwt
**ψ(top) γ(Dis_Arable+PC_Urban) ε(.) *p*(top)**	**282,13**	**0,00**	**9**	**0,24**
ψ(top) γ(Dis_Arable+PC_Green+PC_Urban) ε(.) *p*(top)	282,61	0,48	10	0,19
ψ(top) γ(PC_Urban) ε(.) *p*(top)	282,98	0,85	8	0,15
ψ(top) γ(PC_Green+PC_Urban) ε(.) *p*(top)	283,10	0,97	9	0,15
ψ(top) γ(Dis_Arable+PC_Forest+PC_Urban) ε(.) *p*(top)	283,91	1,78	10	0,10
ψ(top) γ(DEM+Dis_Arable+PC_Urban) ε(.) *p*(top)	284,12	1,99	10	0,09
ψ(top) γ(Dis_Arable+GHII+PC_Urban) ε(.) *p*(top)	284,12	1,99	10	0,09

*(.) indicates parameters that were held constant – no covariates effects

Table S5. Model results for probability of detection (*p*) from dynamic N-mixture models of Ring-necked parakeet in the urban complex of Thessaloniki, Greece, 2024-2025. Initial abundance (λ_1), recruitment (γ), and apparent survival (ω) were held constant in all models. The models are ranked based on the lowest Akaike's Information Criterion (*AIC*) where *deltaAIC* = AIC_i – minimum AIC, *nPars* = number of parameters, and *AICwt* = AIC weight. Only models with deltaAIC < 2 are shown.

Model	AIC	deltaAIC	nPars	AICwt
**λ_1(.) γ(.) ω(.) *p*(.) – NB distribution**	**731,20**	**0,00**	**4**	**0,39**
λ_1(.) γ(.) ω(.) *p*(jday) – NB distribution	732,72	1,52	5	0,18
λ_1(.) γ(.) ω(.) *p*(mas) – NB distribution	732,99	1,79	5	0,16

*(.) indicates parameters that were held constant – no covariates effects

Table S6. Model results for probability of initial abundance (λ_1) from dynamic N-mixture models of Ring-necked parakeet in the urban complex of Thessaloniki, Greece, 2024-2025. The models included the top detection model (null), represented as *p*(top) Recruitment (γ), and apparent survival (ω) were held constant in all models. The models are ranked based on the lowest Akaike's Information Criterion (*AIC*) where *deltaAIC* = AIC_i – minimum AIC, *nPars* = number of parameters, and *AICwt* = AIC weight. Only models with deltaAIC < 2 are shown.

Model	AIC	deltaAIC	nPars	AICwt
**λ_1(PC_Urban+PC_Green+DEM) γ(.) ω(.) *p*(top)**	**674,87**	**0,00**	**7**	**1.00**

*(.) indicates parameters that were held constant – no covariates effects

References

55. European Environmental Agency. Urban Atlas Land Cover/Land Use 2018 (Vector), Europe, 6-Yearly, Jul. 2021; European Envi-ronmental Agency: Copenhagen, Denmark, 2020.
56. European Environmental Agency. Urban Atlas Street Tree Layer 2012 (Vector), Europe, 6-Yearly, May 2016; European Environ-mental Agency: Copenhagen, Denmark, 2023.
57. Sanderson, E.W.; Jaiteh, M.; Levy, M.A.; Redford, K.H.; Wannebo, A.V.; Woolmer, G. The Human Footprint and the Last of the Wild: The Human Footprint Is a Global Map of Human Influence on the Land Surface, Which Suggests That Human Beings Are Stewards of Nature, Whether We like It or Not. Bioscience **2002**, 52, 891–904.
58. European Environmental Agency. European Digital Elevation Model (Raster 25 m), Apr. 2016; European Environmental Agency: Copenhagen, Denmark, 2016.
59. European Environmental Agency Impervious Built-Up 2021 (Raster 10 m), Europe, 3-Yearly, Jun.2025; European Environmental Agency: Copenhagen, Denmark 2025.
60. Geofabrik GmbH. Geofabrik. 2020. https://www.geofabrik.de/en/index.html
